# Epitranscriptomic regulation of endothelial plasticity under hemodynamic forces: insights from the KLF2/4–METTL3–H19 pathway

**DOI:** 10.3389/fgene.2026.1771275

**Published:** 2026-05-29

**Authors:** Jing Huang, Yongyao Yang

**Affiliations:** Department of Cardiology, Guizhou Provincial People’s Hospital, Guiyang, Guizhou, China

**Keywords:** endothelial, H19, KLF2/4, m6A modification, METTL3, plasticity, shear stress

## Abstract

Hemodynamic shear stress is a fundamental biomechanical cue that shapes endothelial phenotypes and contributes to vascular diseases. Although flow-responsive transcription factors such as KLF2 and KLF4 are well recognized, how mechanical forces converge on epitranscriptomic mechanisms to regulate RNA fate remains incompletely understood. Emerging evidence identifies N^6^-methyladenosine (m^6^A) modification as a dynamic regulator of non-coding RNA stability and endothelial function, yet its integration into shear-dependent remodeling has not been systematically explored. This review synthesizes current evidence on the interplay between shear stress and the m^6^A regulatory machinery and discusses how epitranscriptomic modulation of coding and non-coding RNAs may contribute to endothelial plasticity. Within this broader framework, the KLF2/4–METTL3–H19 pathway is presented as a representative mechanistic module illustrating how flow-responsive transcriptional programs may intersect with RNA methylation processes and reader-mediated transcript regulation. The potential implications of such interactions for endothelial-to-mesenchymal transition, barrier integrity, angiogenesis, and vascular diseases—including atherosclerosis, pulmonary arterial hypertension, and diabetic microangiopathy—are discussed. This integrated perspective highlights the emerging role of mechano-epitranscriptomic crosstalk in vascular biology and identifies directions for future investigation.

## 1 Introduction

Hemodynamic shear stress is a fundamental biomechanical signal that governs the spatial and temporal heterogeneity of endothelial cells (ECs) across the vascular system. By sensing the physical properties of blood flow, endothelial cells adapt their phenotype accordingly, playing critical roles in both vascular homeostasis and disease progression (Humphrey and Schwartz, 2021; Davies et al., 2013). Distinct flow patterns—such as laminar shear stress (LSS) and oscillatory shear stress (OSS)—elicit protective or pathological endothelial phenotypes, primarily through flow-sensitive transcription factors like KLF2 and KLF4 (Moonen et al., 2022; Nakajima and Mochizuki, 2017). While the transcriptional programs downstream of shear stress have been partially elucidated, a key unresolved question remains: how mechanical cues are integrated into post-transcriptional regulatory layers—particularly RNA modification pathways—to ultimately determine endothelial cell fate. This gap highlights a critical missing link between well-characterized transcriptional responses and the RNA-level mechanisms that fine-tune endothelial plasticity.

Among emerging RNA-level regulatory mechanisms, N^6^-methyladenosine (m^6^A) has been identified as the most prevalent and dynamic internal RNA modification in eukaryotic cells (Ruden, 2025). m^6^A modulates multiple aspects of RNA metabolism—including splicing, export, stability, localization, and translation—and has been increasingly implicated in cardiovascular and inflammatory disorders (Wang et al., 2023; Chen et al., 2021). In vascular contexts, dysregulated m^6^A signaling has been associated with endothelial inflammation, atherosclerosis, and vascular remodeling (Wang et al., 2023; Chien et al., 2021). However, most current studies focus on individual RNA targets or specific modifying enzymes, without integrating these observations into a broader mechanotransductive framework. In particular, whether hemodynamic forces globally remodel the endothelial m^6^A landscape—and how such remodeling intersects with canonical flow-responsive transcriptional pathways—remains insufficiently defined.

The m^6^A regulatory system operates through coordinated actions of methyltransferase “writers” (e.g., METTL3/METTL14/WTAP), demethylase “erasers” (FTO, ALKBH5), and “reader” proteins, including YTH domain–containing factors and IGF2BP family members, which collectively determine RNA fate (Ruden, 2025; Wang et al., 2023). Emerging evidence suggests that components of this machinery participate in vascular pathophysiology. For example, METTL3-dependent m^6^A deposition has been shown to promote endothelial inflammatory activation and atherogenic dysfunction (Chien et al., 2021), while broader cardiovascular-focused analyses highlight dynamic regulation of writers, erasers, and readers in disease settings (Wang et al., 2023; Chen et al., 2021; Chien et al., 2021). Beyond protein-coding transcripts, m^6^A also regulates long non-coding RNAs (lncRNAs), adding an additional layer of complexity to endothelial gene control. In this regard, METTL3-mediated m^6^A stabilization of the lncRNA H19 has been demonstrated to enhance inflammatory signaling and accelerate atherosclerotic progression in endothelial contexts (Tang et al., 2024). These findings collectively raise the possibility that m^6^A-dependent control of both coding and non-coding RNAs may function as a mechano-responsive regulatory layer within the endothelium.

In this review, we first outline how mechanical stimuli shape endothelial transcriptional programs and summarize current evidence linking hemodynamic forces to the broader m^6^A machinery, encompassing writers, erasers, and readers. We then discuss how m^6^A-mediated regulation of coding and non-coding RNAs contributes to endothelial phenotypic modulation in vascular disease. Within this broader context, we introduce a representative mechanistic module centered on the KLF2/4–METTL3–H19 pathway as a conceptual example of how mechano-epitranscriptomic crosstalk may be organized. In this proposed framework, shear stress–responsive transcription factors are hypothesized to interface with RNA methylation machinery, thereby potentially influencing the stability of m^6^A-modified transcripts through reader competition (e.g., IGF2BP versus YTHDF2), which may contribute to endothelial plasticity.

By situating this focused axis within the wider epitranscriptomic landscape, this review aims to integrate mechanotransduction with RNA-level regulation in a comprehensive and balanced manner. A clearer understanding of how mechanical forces coordinate transcriptional and epitranscriptional layers may provide new insights into endothelial adaptation in health and disease and reveal potential avenues for targeted vascular intervention.

## 2 Shear stress–driven KLF2/4 transcriptional programming

Shear stress is a primary biomechanical signal sensed by ECs, which transduce it into gene regulatory programs that govern their phenotypic plasticity (Nakajima and Mochizuki, 2017). Among the transcriptional mediators of flow-induced responses, Kruppel-like factors 2 and 4 (KLF2 and KLF4) are central regulators of endothelial homeostasis and vascular integrity (Chang and Jain, 2021).

Under physiological conditions, LSS promotes the upregulation of KLF2 and KLF4, leading to the activation of anti-inflammatory, antioxidative, and barrier-enhancing genes such as eNOS (NOS3), THBD, and CLDN5 (Krenning et al., 2016). In contrast, OSS—typically found in disturbed flow regions such as vascular bifurcations—suppresses KLF2/4 expression, thereby facilitating pro-inflammatory signaling, increased permeability, and a shift toward mesenchymal-like phenotypes via processes such as EndMT. This dichotomy in gene expression underlies the spatial heterogeneity of endothelial behavior in vascular health and disease (He et al., 2022).

The sensitivity of KLF2/4 to shear stress is mediated by a network of upstream mechanosensors. The PECAM1–VE-cadherin–VEGFR2 complex acts as a canonical signal transduction hub that relays extracellular mechanical forces into intracellular signaling cascades, including Src family kinases (Tamargo et al., 2023). Additionally, mechanosensitive ion channels such as Piezo1 respond to shear-induced membrane deformation by triggering calcium influx and downstream activation of KLF2/4 transcription (Cheng et al., 2025). Chromatin accessibility at enhancer regions is also dynamically remodeled in response to flow, further modulating the spatial and temporal expression of KLF factors (Ku et al., 2019).

Recent studies, however, indicate that flow-induced regulation of KLF2 and KLF4 is not entirely consistent across experimental systems. Moonen et al. reported robust KLF4 induction under laminar shear stress (Moonen et al., 2022), whereas Nakajima et al. observed a more modest or delayed response under similar shear profiles, suggesting that KLF4 may function partly as a secondary rather than immediate flow-responsive factor (Nakajima and Mochizuki, 2017). Such discrepancies may arise from differences in shear magnitude, exposure duration, endothelial subtype, or chromatin accessibility. Similar variability is seen with Piezo1, which responds strongly to high-magnitude or pulsatile shear in some models but shows limited activation under physiological laminar flow, raising debate over whether Piezo1 acts as a universal or context-dependent mechanosensor (Cheng et al., 2025; Baratchi et al., 2020). Together, these variations underscore the need for an integrated framework to explain how distinct flow patterns and mechanosensory pathways converge on KLF2/4 activation.

While KLF2/4 are classically known for regulating protein-coding genes, emerging evidence suggests they may also influence epigenetic and epitranscriptomic programs. For instance, KLF4 has been shown to interact with chromatin modifiers such as HDACs and p300, suggesting a broader regulatory role that may extend to RNA-modifying enzymes (Bure et al., 2022; Tomé and Almeida, 2024). Based on these insights, we propose a testable hypothesis: KLF2/4 may regulate the expression, localization, or catalytic activity of the m^6^A methyltransferase METTL3, thus serving as a conduit through which mechanical forces reprogram the epitranscriptome.

## 3 Mechanical regulation of the m^6^A epitranscriptomic machinery

Beyond transcriptional reprogramming, accumulating evidence indicates that hemodynamic forces (particularly laminar vs. disturbed/oscillatory flow) reshape the endothelial m6A landscape, while other mechanical cues (e.g., matrix stiffness and cyclic stretch) have been implicated in m6A regulation in mechanosensitive contexts and may be relevant during vascular remodeling (Li et al., 2025; Li et al., 2022). Laminar and oscillatory shear stress have been shown to differentially modulate the expression and activity of m^6^A writers, including METTL3 and potentially other writer components, thereby altering RNA methylation profiles in a flow-dependent manner (Zhao et al., 2025). Genome-wide m^6^A mapping studies further reveal that disturbed flow induces transcript-specific methylation changes in endothelial cells, which are associated with inflammatory gene activation (Li et al., 2022).

Importantly, m^6^A dynamics are governed not only by methyltransferases but also by demethylases. Emerging data suggest that FTO and ALKBH5 expression can be influenced by inflammatory and metabolic cues that frequently coexist with abnormal hemodynamic forces, implying that mechanical stress may shift the balance between methylation and demethylation processes (Shan et al., 2024; Gao et al., 2024).

In addition to writers and erasers, m^6^A reader proteins—including YTH domain-containing factors and IGF2BP family members—determine RNA stability, translation efficiency, and subcellular localization (Zou and He, 2024; Liu et al., 2025). Although direct evidence linking mechanical forces to reader redistribution remains limited, flow-induced changes in RNA-binding protein expression suggest that biomechanical signals may regulate not only the installation of m^6^A marks but also their functional interpretation (Li et al., 2024).

Collectively, these observations support the concept that mechanical stimuli engage the m^6^A regulatory network at multiple levels, providing a multilayered mechanism through which hemodynamic forces influence endothelial phenotype.

## 4 From transcription to epitranscription—METTL3-mediated m^6^A installation and H19 fate

Beyond the classical DNA–transcription–protein axis, RNA modifications have emerged as a critical regulatory layer in determining cell fate (Luan et al., 2025). Among these, m^6^A is the most prevalent and dynamically regulated internal modification in eukaryotic mRNA and long non-coding RNAs (lncRNAs) (Chien et al., 2021). In ECs, however, the role of m^6^A in fine-tuning gene expression in response to hemodynamic cues is only beginning to be explored (Li et al., 2022).

The m^6^A writer complex—centered on METTL3 and supported by METTL14 and WTAP—installs methylation marks that can be removed by FTO and ALKBH5 and interpreted by reader proteins such as IGF2BP and YTHDF families (Liang et al., 2020; Pagiatakis and Condorelli, 2019; Feng et al., 2023; Zhang et al., 2021). Strong evidence supports a direct role of METTL3 in regulating H19 stability, as Tang et al. demonstrated that METTL3-mediated m^6^A enhances H19 stability and promotes endothelial inflammatory activation (Tang et al., 2024). Moderate evidence further suggests that disturbed flow alters vascular m^6^A patterns, as shown by Li et al. in arteries exposed to oscillatory shear stress (Li et al., 2022). Moreover, H19 contains multiple conserved m^6^A motifs, and its m^6^A-dependent regulation of stability and localization has been demonstrated in vascular and non-vascular contexts (Tang et al., 2024; Zhang et al., 2025).

In contrast, evidence directly linking KLF2/4 to METTL3 remains limited. Although KLF4 interacts with chromatin modulators such as HDACs and p300 (Bure et al., 2022; Tomé and Almeida, 2024), whether KLF2/4 transcriptionally activates METTL3 or modulates its localization under shear stress has not been experimentally verified. Therefore, the proposed KLF2/4–METTL3 connection should be regarded as a mechanistic hypothesis supported primarily by indirect evidence rather than a validated pathway.

At present, several non-mutually exclusive regulatory scenarios can be envisioned. One possibility is direct transcriptional control, whereby KLF2/4 could potentially engage regulatory elements within the METTL3 promoter or enhancer regions under defined shear conditions (He et al., 2022; Souilhol et al., 2020). Alternatively, KLF2/4 may influence METTL3 indirectly through intermediate signaling cascades, chromatin remodeling complexes, or metabolic regulators that modulate methyl-donor availability and epitranscriptomic enzyme activity (He and He, 2021). Distinguishing between direct promoter occupancy and indirect regulatory circuits will require dedicated experimental validation.

Reader competition provides a mechanistic basis for how m^6^A modifications alter H19 fate. IGF2BP proteins stabilize m^6^A-modified transcripts (Huang et al., 2018), whereas YTHDF2 recruits decay machinery to promote degradation (Ru et al., 2025). This antagonistic reader competition creates an m^6^A-dependent switch that determines whether H19 is stabilized or degraded under distinct shear conditions, thereby linking mechanical cues to RNA fate decisions (Wei, 2024).

Importantly, the outcome of this reader competition is unlikely to be purely stochastic and may be shaped by multiple regulatory layers. Local protein stoichiometry—reflecting the relative abundance and turnover of IGF2BP and YTHDF2 across different cellular contexts—can bias binding probability toward stabilization or decay (Huang et al., 2018; Wei, 2024; Wang et al., 2014). In addition, post-translational modifications of reader proteins, such as phosphorylation, may alter their RNA-binding affinity, subcellular localization, or interaction with decay and stabilization complexes, thereby influencing competitive occupancy on m6A-marked H19 (Hou et al., 2021; Hornegger et al., 2024; Zaccara et al., 2019). Subcellular compartmentalization and coupling to ribonucleoprotein assemblies further add contextual control (Zaccara and Jaffrey, 2020; Ries et al., 2019). These determinants collectively suggest that disturbed-flow–associated signaling pathways may contribute to re-weighting reader competition rather than merely switching the pathway on or off.

Future studies—such as KLF2/4 ChIP-seq under defined shear patterns, METTL3 promoter reporter assays, and MeRIP-seq or CRISPR-based demethylation of H19 m^6^A sites under LSS versus OSS—are required to determine whether mechanical forces truly remodel the m^6^A landscape of H19 and solidify this proposed epitranscriptomic axis.

Beyond experimental validation, it is equally important to consider how this proposed module behaves in atheroprone regions exposed to disturbed or oscillatory shear stress. In such environments, canonical KLF2/4-dependent atheroprotective programs are attenuated, raising the possibility that the KLF2/4–METTL3–H19 axis may be either suppressed or functionally rewired (He et al., 2022; Souilhol et al., 2020; Chiu and Chien, 2011). One scenario is “axis attenuation,” in which reduced KLF2/4 activity may diminish support for METTL3 expression or activity, thereby weakening m^6^A-dependent stabilization of select transcripts. Alternatively, disturbed flow–associated inflammatory signaling, oxidative stress, and metabolic remodeling may modulate writers, erasers, or reader proteins independently of KLF2/4, potentially redirecting m6A-mediated RNA fate decisions and shifting reader competition (e.g., IGF2BP versus YTHDF2) toward atheroprone transcriptomic programs (Tamargo et al., 2023; Wei, 2024). These context-dependent mechanisms suggest that the epitranscriptomic output of this module may differ substantially between laminar and disturbed flow conditions, which may provide a conceptual framework for understanding endothelial dysfunction in disease-prone vascular regions.

## 5 Broader m6A targets of METTL3 in endothelial biology

While H19 provides a tractable model for illustrating m6A-dependent RNA fate control within the proposed shear-responsive framework, it is important to recognize that METTL3 regulates a diverse repertoire of endothelial transcripts. For example, METTL3-dependent m6A signaling has been implicated in inflammatory activation of lung endothelial cells, including regulation of adhesion molecules (e.g., ICAM1) (Feng et al., 2025). In addition, METTL3 has been shown to modulate angiogenesis through m6A-dependent control of angiogenic microRNAs, suggesting broader involvement in vascular remodeling (Chamorro-Jorganes et al., 2021). Together, these studies support the view that METTL3 can act as a broad epitranscriptomic regulator in endothelial cells, rather than being confined to a single downstream transcript (Qin et al., 2020).

Notably, these METTL3 substrates encompass distinct RNA classes and regulatory modes. Protein-coding mRNAs often mediate m6A-dependent changes in transcript stability or translation (Zou and He, 2024; Zaccara and Jaffrey, 2020), whereas microRNAs influence angiogenic networks through coordinated post-transcriptional regulation (Chamorro-Jorganes et al., 2021). In contrast, lncRNAs such as H19 may serve as integrative nodes linking m6A-dependent stability control with RNA–protein interaction networks, consistent with emerging evidence that m6A reader proteins can participate in RNA condensate formation (Wang et al., 2020). This comparison clarifies why H19 can be used as a representative “molecular switch,” while underscoring that METTL3-dependent endothelial phenotypes likely emerge from multi-target m6A programs rather than a single downstream transcript.

Within this broader landscape, H19 can be viewed as a representative mechanosensitive substrate that illustrates how reader competition may translate m6A marks into context-dependent endothelial phenotypes. Expanding future studies to include additional METTL3 targets will be essential for determining whether the proposed shear stress–epitranscriptomic axis represents a transcript-specific mechanism or part of a more extensive regulatory network.

## 6 H19 as a representative m6A-Regulated node in endothelial plasticity and disease linkage

Building on the broader landscape of METTL3-dependent regulation described above, we next focus on H19 as a representative mechanosensitive node. Within the proposed shear stress–KLF2/4–METTL3 axis, H19 is positioned to undergo dynamically regulated m^6^A modifications, placing it as a pivotal node that links epitranscriptomic changes to functional endothelial phenotypes (Liu Z.-Y. et al., 2024; Chen et al., 2024). This section focuses on the phenotypic consequences of H19 regulation, particularly its influence on three key aspects of endothelial plasticity, and connects these outputs to disease contexts such as atherosclerosis (AS), pulmonary arterial hypertension (PAH), and diabetic microvascular complications, thereby highlighting its translational potential.

One major manifestation of endothelial plasticity is the EndMT, a process in which cells lose endothelial markers (e.g., CDH5) and acquire mesenchymal features (e.g., VIM, SNAI1), resulting in compromised barrier function, increased motility, and pathological remodeling (Yoshimatsu and Watabe, 2022; Derada Troletti et al., 2019). H19 has been implicated in EndMT regulation through multiple mechanisms, including functioning as a competitive endogenous RNA (ceRNA) and mediating m^6^A-dependent stability control (Tang et al., 2024; Yu et al., 2024). We propose that shear stress–induced remodeling of H19’s m^6^A profile alters its RNA stability, thereby modulating the initiation and progression of EndMT. This effect may dynamically counterbalance the anti-EndMT roles of KLF2/4 in maintaining endothelial quiescence.

The barrier function of endothelial cells, a core determinant of vascular homeostasis, relies on the integrity of tight junction proteins (e.g., CLDN5, ZO-1) and the glycocalyx layer (Richard et al., 2022). H19 has been shown to influence glycocalyx-modifying enzymes and regulate cytoskeletal rearrangement, affecting intercellular junction stability. In settings of disturbed flow and increased permeability, altered H19 expression and stability correlate with electrical resistance (TEER) measurements (Liu X. et al., 2024; Sukumar, 2020). This suggests that m^6^A-mediated control of H19 may serve as a buffer against barrier disruption under mechanical stress.

Angiogenesis, another hallmark of endothelial plasticity, is modulated by H19 through mechanisms involving nitric oxide (NO) bioavailability, tip/stalk cell fate determination, and VEGF signaling (Luan et al., 2025; Shirvaliloo, 2023; Subramaniam, 2022). In ischemic or inflammatory environments, H19 expression levels correlate with sprouting density and migratory behavior (Huang et al., 2024). We propose that shear-sensitive m^6^A stabilization of H19 may modulate its angiogenic potential over time, offering a post-transcriptional tuning mechanism that aligns vascular growth with flow conditions.

These three axes of plasticity—EndMT, barrier integrity, and angiogenesis—not only reflect endothelial adaptability but also embed H19 into the pathogenesis of several vascular diseases: In atherosclerosis, reduced H19 stability in disturbed-flow regions may promote EndMT and weaken barrier function (Tamargo et al., 2023); In PAH, H19 contributes to pulmonary microvascular remodeling and abnormal angiogenesis (Dou et al., 2024); In diabetic microangiopathy, altered shear stress and hyperglycemia impact the m^6^A-dependent stability of H19, affecting vascular permeability and repair (Liu X. et al., 2024).

**FIGURE 1:**
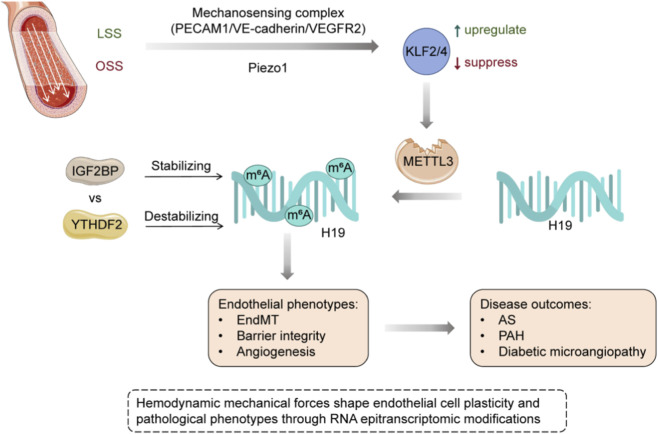
H19 as a central regulator of endothelial plasticity.

Together, these findings position H19 not just as a passive RNA target but as an active integrator of shear stress, epitranscriptomic modification, and phenotypic output. Its post-transcriptional fate—governed by m^6^A methylation and reader protein competition—translates upstream mechanical and transcriptional cues into downstream cellular behaviors. Figure 1 summarizes this model, illustrating how H19 connects shear-mediated m^6^A regulation to plastic phenotypes and disease-specific vascular contexts.

## 7 Conclusion

Shear stress represents a fundamental biomechanical determinant of endothelial cell identity and phenotypic plasticity. However, the mechanisms by which mechanical cues are integrated into RNA-level regulatory programs remain incompletely understood. In this review, we propose a conceptual mechano-epitranscriptomic framework—“Shear stress → KLF2/4 → METTL3 → H19 → m^6^A reader competition → Endothelial plasticity”—to bridge transcriptional mechanotransduction with post-transcriptional RNA fate control. Within this model, laminar flow–responsive KLF2/4 are hypothesized to interface with METTL3-dependent m^6^A deposition, thereby influencing the stability and functional output of the long non-coding RNA H19 through context-dependent reader competition (e.g., IGF2BP versus YTHDF2). Rather than functioning as a simple binary switch, this regulatory node may dynamically tune endothelial phenotypes—including EndMT, barrier integrity, and angiogenic behavior—across distinct hemodynamic environments.

To rigorously evaluate this framework, multi-layered experimental approaches will be required. Chromatin immunoprecipitation sequencing (ChIP-seq) under defined shear conditions can assess potential KLF2/4 occupancy at METTL3 regulatory regions. Time-resolved MeRIP-seq or m6A-seq, coupled with RNA stability assays, may delineate flow-dependent methylation dynamics. Site-specific CRISPR-based m^6^A editing strategies can provide causal validation of methylation-dependent transcript fate, while functional assays such as TEER, permeability analysis, and angiogenic sprouting assays will link molecular events to phenotypic outputs. Integration with single-cell and spatial multi-omics platforms will further enable resolution of endothelial heterogeneity in atheroprotective versus atheroprone regions.

Beyond mechanistic clarification, this axis highlights potential translational opportunities. Shear-sensitive m^6^A signatures on H19 or related transcripts may serve as biomarkers of endothelial dysregulation, whereas modulation of reader competition could represent a novel strategy to restore RNA homeostasis. Nevertheless, challenges remain, including modification specificity, flow-pattern heterogeneity, and long-term safety considerations. Continued advances in programmable RNA editing, spatial epitranscriptomics, and vascular-targeted delivery systems will be essential to transform this conceptual framework into actionable therapeutic strategies.
